# 2-Methyl­piperidinium bromide

**DOI:** 10.1107/S1600536812022878

**Published:** 2012-05-31

**Authors:** Qian Xu

**Affiliations:** a Ordered Matter Science Research Center, Southeast University, Nanjing 211189, People’s Republic of China

## Abstract

In the title organic–inorganic hybrid salt, C_6_H_14_N^+^·Br^−^, N—H⋯Br hydrogen bonds link the cations and anions, forming extended hydrogen-bonded chains along the *c* axis.

## Related literature

For general background to ferroelectric organic frameworks, see: Ye *et al.* (2006 ▶); Zhang *et al.* (2008 ▶, 2010 ▶).
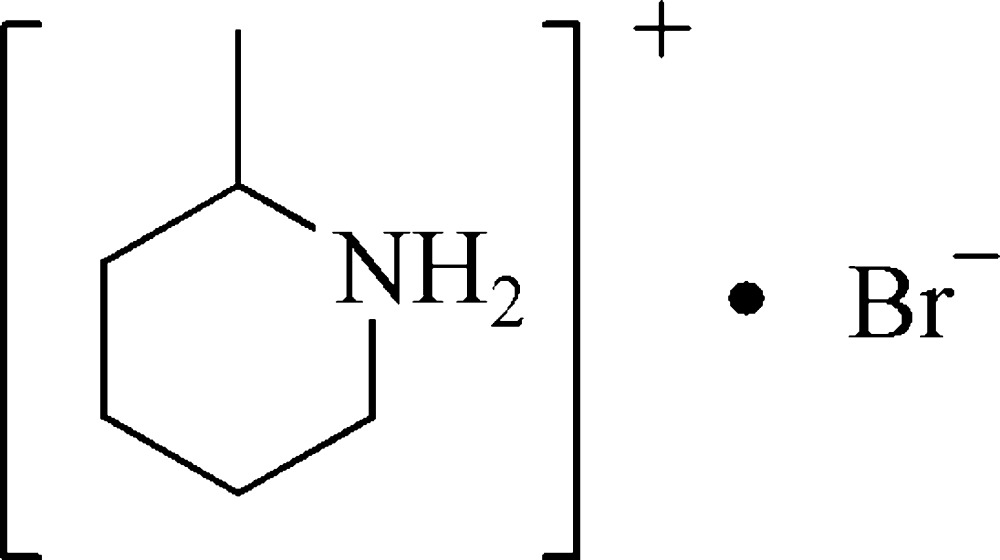


## Experimental

### 

#### Crystal data

C_6_H_14_N^+^·Br^−^
*M* *_r_* = 180.09
Orthorhombic, 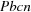

*a* = 22.137 (4) Å
*b* = 9.918 (2) Å
*c* = 7.5853 (15) Å
*V* = 1665.5 (6) Å^3^
*Z* = 8
Mo *K*α radiation
μ = 4.85 mm^−1^
*T* = 293 K
0.55 × 0.44 × 0.36 mm

#### Data collection

Rigaku SCXmini diffractometer
Absorption correction: multi-scan (*CrystalClear*; Rigaku, 2005 ▶) *T*_min_ = 0.134, *T*_max_ = 0.223
15678 measured reflections
1907 independent reflections
1142 reflections with *I* > 2σ(*I*)
*R*_int_ = 0.109

#### Refinement

*R*[*F* ^2^ > 2σ(*F* ^2^)] = 0.049
*wR*(*F* ^2^) = 0.118
*S* = 1.05
1907 reflections
75 parameters
H-atom parameters constrained
Δρ_max_ = 0.38 e Å^−3^
Δρ_min_ = −0.48 e Å^−3^

### 

Data collection: *SCXmini* (Rigaku, 2006 ▶); cell refinement: *SCXmini*; data reduction: *SCXmini*; program(s) used to solve structure: *SHELXS97* (Sheldrick, 2008 ▶); program(s) used to refine structure: *SHELXL97* (Sheldrick, 2008 ▶); molecular graphics: *DIAMOND* (Brandenburg & Putz, 2005 ▶); software used to prepare material for publication: *SHELXL97*.

## Supplementary Material

Crystal structure: contains datablock(s) I, global. DOI: 10.1107/S1600536812022878/fy2056sup1.cif

Structure factors: contains datablock(s) I. DOI: 10.1107/S1600536812022878/fy2056Isup2.hkl

Supplementary material file. DOI: 10.1107/S1600536812022878/fy2056Isup3.cml

Additional supplementary materials:  crystallographic information; 3D view; checkCIF report

## Figures and Tables

**Table 1:** Hydrogen-bond geometry (Å, °)

*D*—H⋯*A*	*D*—H	H⋯*A*	*D*⋯*A*	*D*—H⋯*A*
N1—H1*A*⋯Br1	0.90	2.34	3.238 (4)	176
N1—H1*B*⋯Br1^i^	0.90	2.36	3.262 (3)	176

Symmetry code: (i) 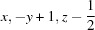.

**Fig. 1.**
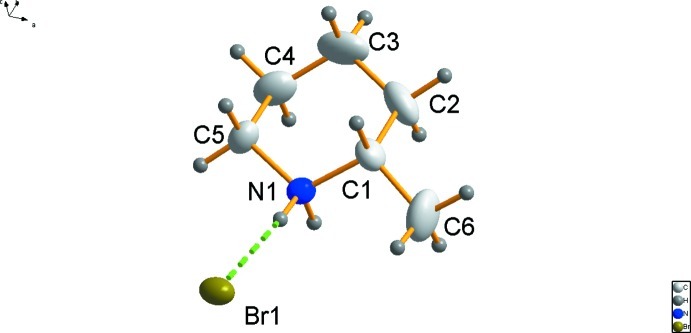
Molecular structure of the title compound, with the atomic numbering scheme. Displacement ellipsoids are drawn at the 30% probability level. The dashed line indicates a hydrogen bond.

**Fig. 2.**
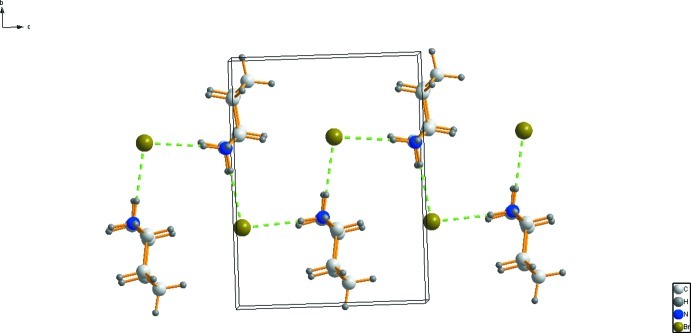
A view of the packing of the title compound along the a axis. Dashed lines indicate hydrogen bonds.

## supplementary crystallographic information

### Comment

Dielectric-ferroelectrics constitute an interesting class of materials, comprising organic ligands,metal-organic coordination compounds and organic-inorganic hybrids.(Zhang *et al.*, 2010; Zhang *et al.*, 2008; Ye *et al.*, 2006). Unfortunately,the dielectric constant of the title compound as a function of temperature indicates that the permittivity is basically temperature-independent below the melting point of the compound (428-429K). We have found that title compound has no dielectric disuniformity from 80 K to 405 K. Herein we descibe the crystal structure of this compound.

Regarding its crystal structure, the asymmetric unit of the title compound consists of a 2-methylpiperidinium cation and a bromide anion (Fig. 1). The cations and anions are connected by N—H···Br hydrogen bonds, which make a great contribution to the stability of the crystal structure (Fig. 2 and Table 1).

### Experimental

The title compound was obtained by the addition of hydrobromic acid (0.8 g, 0.01 mol) to a solution of 2-methylpiperidine (0.97 g, 0.01 mol) in water, *i.e.*, in the stoichiometric ratio of 1:1. Good quality single crystals were obtained by slow evaporation of water after two days (the chemical yield is 65%).

### Refinement

All H atoms were placed in geometrically idealized positions and constrained to ride on their parent atoms with C—H = 0.97–0.98 Å, N—H = 0.90 Å and with *U*_iso_(H) = 1.2*U*_iso_(C, N) and *U*_iso_(H) = 1.5*U*_iso_(C) for methyl hydrogen atoms.

### Crystal data

**Table:**

C_6_H_14_N^+^·Br^−^	*F*(000) = 736
*M**_r_* = 180.09	*D*_x_ = 1.436 Mg m^−^^3^
Orthorhombic, *P**b**c**n*	Mo *K*α radiation, λ = 0.71073 Å
Hall symbol: -P 2n 2ab	Cell parameters from 3638 reflections
*a* = 22.137 (4) Å	θ = 3.0–27.5°
*b* = 9.918 (2) Å	µ = 4.85 mm^−^^1^
*c* = 7.5853 (15) Å	*T* = 293 K
*V* = 1665.5 (6) Å^3^	Block, colorless
*Z* = 8	0.55 × 0.44 × 0.36 mm

### Data collection

**Table:**

Rigaku SCXmini diffractometer	1907 independent reflections
Radiation source: fine-focus sealed tube	1142 reflections with *I* > 2σ(*I*)
Graphite monochromator	*R*_int_ = 0.109
Detector resolution: 13.6612 pixels mm^-1^	θ_max_ = 27.5°, θ_min_ = 3.4°
ω scans	*h* = −28→28
Absorption correction: multi-scan (*CrystalClear*; Rigaku, 2005)	*k* = −12→12
*T*_min_ = 0.134, *T*_max_ = 0.223	*l* = −9→9
15678 measured reflections	

### Refinement

**Table:**

Refinement on *F*^2^	Secondary atom site location: difference Fourier map
Least-squares matrix: full	Hydrogen site location: inferred from neighbouring sites
*R*[*F*^2^ > 2σ(*F*^2^)] = 0.049	H-atom parameters constrained
*wR*(*F*^2^) = 0.118	*w* = 1/[σ^2^(*F*_o_^2^) + (0.0407*P*)^2^] where *P* = (*F*_o_^2^ + 2*F*_c_^2^)/3
*S* = 1.05	(Δ/σ)_max_ = 0.001
1907 reflections	Δρ_max_ = 0.38 e Å^−^^3^
75 parameters	Δρ_min_ = −0.48 e Å^−^^3^
0 restraints	Extinction correction: *SHELXL97* (Sheldrick, 2008), Fc^*^=kFc[1+0.001xFc^2^λ^3^/sin(2θ)]^-1/4^
Primary atom site location: structure-invariant direct methods	Extinction coefficient: 0.0022 (5)

### Special details

**Table:**

Geometry. All e.s.d.'s (except the e.s.d. in the dihedral angle between two l.s. planes) are estimated using the full covariance matrix. The cell e.s.d.'s are taken into account individually in the estimation of e.s.d.'s in distances, angles and torsion angles; correlations between e.s.d.'s in cell parameters are only used when they are defined by crystal symmetry. An approximate (isotropic) treatment of cell e.s.d.'s is used for estimating e.s.d.'s involving l.s. planes.
Refinement. Refinement of *F*^2^ against ALL reflections. The weighted *R*-factor *wR* and goodness of fit *S* are based on *F*^2^, conventional *R*-factors *R* are based on *F*, with *F* set to zero for negative *F*^2^. The threshold expression of *F*^2^ > σ(*F*^2^) is used only for calculating *R*-factors(gt) *etc*. and is not relevant to the choice of reflections for refinement. *R*-factors based on *F*^2^ are statistically about twice as large as those based on *F*, and *R*- factors based on ALL data will be even larger.

### Fractional atomic coordinates and isotropic or equivalent isotropic displacement parameters (Å^2^)

**Table:**

	*x*	*y*	*z*	*U*_iso_*/*U*_eq_	
C1	0.6672 (2)	0.7098 (5)	0.0449 (6)	0.0546 (13)	
H1	0.6680	0.6902	0.1715	0.065*	
C2	0.6608 (3)	0.8597 (6)	0.0205 (7)	0.0817 (19)	
H2A	0.6633	0.8808	−0.1041	0.098*	
H2B	0.6939	0.9049	0.0797	0.098*	
C3	0.6018 (3)	0.9124 (6)	0.0922 (8)	0.097 (2)	
H3A	0.6007	0.8994	0.2189	0.117*	
H3B	0.5988	1.0082	0.0686	0.117*	
C4	0.5503 (3)	0.8414 (6)	0.0099 (7)	0.0799 (18)	
H4A	0.5128	0.8733	0.0615	0.096*	
H4B	0.5494	0.8611	−0.1153	0.096*	
C5	0.5556 (2)	0.6950 (5)	0.0365 (6)	0.0594 (13)	
H5A	0.5226	0.6497	−0.0231	0.071*	
H5B	0.5528	0.6747	0.1613	0.071*	
C6	0.7220 (2)	0.6499 (6)	−0.0376 (8)	0.104 (2)	
H6A	0.7210	0.5536	−0.0242	0.156*	
H6B	0.7575	0.6850	0.0189	0.156*	
H6C	0.7229	0.6722	−0.1607	0.156*	
N1	0.61363 (14)	0.6448 (4)	−0.0327 (4)	0.0436 (9)	
H1A	0.6158	0.5554	−0.0128	0.052*	
H1B	0.6143	0.6574	−0.1502	0.052*	
Br1	0.61324 (2)	0.32302 (5)	0.03937 (6)	0.0540 (2)	

### Atomic displacement parameters (Å^2^)

**Table:**

	*U*^11^	*U*^22^	*U*^33^	*U*^12^	*U*^13^	*U*^23^
C1	0.055 (3)	0.060 (3)	0.049 (3)	−0.010 (2)	−0.014 (2)	0.001 (2)
C2	0.100 (5)	0.071 (4)	0.075 (4)	−0.040 (4)	−0.030 (4)	0.017 (3)
C3	0.160 (7)	0.043 (3)	0.088 (5)	0.015 (4)	0.003 (5)	−0.002 (3)
C4	0.095 (5)	0.062 (4)	0.082 (4)	0.027 (3)	0.012 (3)	0.004 (3)
C5	0.050 (3)	0.060 (3)	0.068 (3)	0.008 (2)	0.013 (2)	0.003 (3)
C6	0.046 (4)	0.143 (6)	0.121 (6)	0.003 (3)	0.000 (3)	0.027 (4)
N1	0.048 (2)	0.041 (2)	0.042 (2)	0.0030 (16)	0.0024 (18)	0.0000 (16)
Br1	0.0752 (4)	0.0440 (3)	0.0427 (3)	0.0012 (2)	−0.0015 (2)	−0.0005 (2)

### Geometric parameters (Å, º)

**Table:**

C1—N1	1.473 (5)	C4—H4A	0.9700
C1—C6	1.488 (7)	C4—H4B	0.9700
C1—C2	1.504 (7)	C5—N1	1.475 (5)
C1—H1	0.9800	C5—H5A	0.9700
C2—C3	1.507 (8)	C5—H5B	0.9700
C2—H2A	0.9700	C6—H6A	0.9600
C2—H2B	0.9700	C6—H6B	0.9600
C3—C4	1.478 (8)	C6—H6C	0.9600
C3—H3A	0.9700	N1—H1A	0.9000
C3—H3B	0.9700	N1—H1B	0.9000
C4—C5	1.471 (6)		
			
N1—C1—C6	108.2 (4)	C5—C4—H4B	109.5
N1—C1—C2	107.9 (4)	C3—C4—H4B	109.5
C6—C1—C2	114.9 (4)	H4A—C4—H4B	108.1
N1—C1—H1	108.6	C4—C5—N1	110.7 (4)
C6—C1—H1	108.6	C4—C5—H5A	109.5
C2—C1—H1	108.6	N1—C5—H5A	109.5
C1—C2—C3	112.4 (4)	C4—C5—H5B	109.5
C1—C2—H2A	109.1	N1—C5—H5B	109.5
C3—C2—H2A	109.1	H5A—C5—H5B	108.1
C1—C2—H2B	109.1	C1—C6—H6A	109.5
C3—C2—H2B	109.1	C1—C6—H6B	109.5
H2A—C2—H2B	107.9	H6A—C6—H6B	109.5
C4—C3—C2	110.5 (5)	C1—C6—H6C	109.5
C4—C3—H3A	109.5	H6A—C6—H6C	109.5
C2—C3—H3A	109.5	H6B—C6—H6C	109.5
C4—C3—H3B	109.5	C1—N1—C5	114.3 (4)
C2—C3—H3B	109.5	C1—N1—H1A	108.7
H3A—C3—H3B	108.1	C5—N1—H1A	108.7
C5—C4—C3	110.5 (5)	C1—N1—H1B	108.7
C5—C4—H4A	109.5	C5—N1—H1B	108.7
C3—C4—H4A	109.5	H1A—N1—H1B	107.6

### Hydrogen-bond geometry (Å, º)

**Table:**

*D*—H···*A*	*D*—H	H···*A*	*D*···*A*	*D*—H···*A*
N1—H1*A*···Br1	0.90	2.34	3.238 (4)	176
N1—H1*B*···Br1^i^	0.90	2.36	3.262 (3)	176

Symmetry code: (i) *x*, −*y*+1, *z*−1/2.
